# Notes and Comments

**Published:** 1894-03

**Authors:** 


### Notes and Comments.[1]

PRESENCE OF MIND IN APPLYING ANTIDOTES.—An instance demonstrating the value of presence of mind in emergencies occurred recently in Sag Harbor, N. Y., a report of which may be found in the *Scientific American.*

The little daughter of Dr. Sterling while playing about the house found a bottle which had formally contained citrate of magnesia, and still bore that label. The child took a large swallow. With a scream she dropped the bottle and began to clutch her throat in an agony of pain. Her father, who had heard her screams, found that what the little one had taken for citrate of magnesia was oxalic acid. Seeing that not a moment was to be lost, if he wished to save the child's life, the doctor looked about for an alkaline antidote. Seizing his penknife he sprang to the white-washed wall and scraped some of the lime into his hand. This he threw into a glass partly filled with water, and poured the mixture down the child's throat. The antidote took effect at once. The intense pain caused by the burning acid was alleviated, and soothing, mucilaginous drinks to cool the blistered mouth and throat did the rest.

This expediency has been previously recommended by us when writing upon the subject of emergencies.[2]

---

OVERWORK.—A few possibly die early from overwork, says a well-known writer, but many more pass away from want of enough. Active, brainy men who have not abused their bodily system by excesses are among the longest livers. Gladstone, the English Premier, who is passionately fond of gardening and forestry as a relaxation, and who has been a wonderful worker, is now in his eighty-third year. Also in his eighty-third year is Professor Babington, the professor of botany in Cambridge, England, author of

---

[1] The assistant editor solicits contributions for this department,—new methods, new remedies and formulas, or any short practical note which may prove of value to the practitioner or student. Address 1506 Arch Street, Philadelphia.

[2] See chapter on "Emergencies," composed of dental pathology and dental medicine, published by P. Blakiston, Son & Co., Philadelphia.

one of the best works on the British flora, and a tremendous worker. Another heavy worker is the great American geologist, Professor Hall, of Albany, N. Y., who, though over eighty, was working as actively as a young man at a recent meeting at Rochester. We could fill pages with similar instances, illustrating the fact that hard work does not necessarily mean short life. At any rate, it has always been our opinion that it was much better for us to wear out than to rust out.

---

Professional Etiquette.—We have read with interest in the December issue of the *Dental Review* an editorial by Dr. Johnson upon professional etiquette. He writes particularly of one phase of this subject which we do not remember having seen discussed, but which is of sufficient importance to merit the pages devoted to it. This pertains to the courtesy due a dentist who calls on a brother practitioner during his office-hours, and conversely to the consideration due the practitioner from the caller. It is filled with suggestions that would be valuable to nearly every young practitioner, and to some of the older ones.

---

College Advertising.—It is announced that one of the many Chicago dental colleges has shown so great a lack of self-respect as to have the following advertisement placed upon the back of a menu in a cheap restaurant: "Go to the operating-parlor of the ——— College of Dental Surgery, on floor above; your teeth will receive careful and proper attention at a very moderate cost."

This must have given our foreign friends a singular idea of the college ethics prevailing in that city. When a college that is represented in the National Association of Dental College Faculties shows no more regard for professional interest, we think this Association should take some decided action along these lines.

---

Self-Teaching.—By education is meant the training of the head and heart to the highest attainable degree of perfection and usefulness. A question that is often forced upon the thoughtful mind in this relation is, Can one overcome temperament? Whenever this experiment has been earnestly and thoughtfully tried, it is admitted that inherited traits can be modified, if not entirely overcome. A recent writer in *The Outlook* says, "Temper, disagreeable voice or gesture, an ungraceful walk, a tendency to untruthfulness, lack of confidence, all traits that weaken or mar character, are being constantly effaced by those who recognize inherited burdens. If this were not so, what would we mean by development of character?

"Temperament may be defined as a constitutional organization, or mental and physical traits, depending primarily upon heredity. Now, to reach the highest human perfection—which endeavor is a duty incumbent upon every one—we must study our own, our whole nature; work to overcome every weakness; leave our failures always behind us, and strive to develop, broaden, and elevate our own intelligence. Remember that the estimate of a man, and relatively that of the profession he represents, is usually measured by the breadth of intellect he displays in his intercourse with the world."

---

INFALLIBILITY.—Dr. Welch, in writing editorially in the *Items of Interest*, says truly that it will not do to ever appear too self-confident and infallible. Such an action appears too much like a mask put on to hide weakness and incapacity; neither will it do to express frequent doubts and fears. To distrust one's self is to court the distrust of one's patients. A prompt, manly, straightforward course is quite sufficient to show we know what we are about; that will win confidence, though all men know all men to be fallible. Yet, where results are necessarily in doubt, we should show hesitancy and express our honest conviction. "Why did you not tell me I might have trouble?" from a returning patient, is always embarrassing.

---

AN OBTUNDENT FOR SENSITIVE DENTINE.—As a satisfactory "obtundent" for sensitive dentine is very desirable, I send you herein a formula which has responded more frequently in desired results than any other combination I have used, not excepting my experiments with the secret nostrums sold for the purpose. While this in preparation cost less than one-fourth of those, I have the satisfaction of knowing the constituents, and would advise those who desire an obtundent for this purpose to try this combination before purchasing any secret product.

℞ Cocaine, grs. v;

Carbolic acid, grs. xx;

Chloroform, ʒss;

Muriatic acid, ♏x;

Alcohol, ʒii.

C. N. PEIRCE.

